# Radiobiological and Clinical Advantages of Proton Therapy in Modern Cancer Treatment

**DOI:** 10.3390/cancers18050885

**Published:** 2026-03-09

**Authors:** Spyridon A. Kalospyros, Angeliki Gkikoudi, Athanasios Koutsostathis, Athanasia Adamopoulou, Spyridon N. Vasilopoulos, Vasileios Rangos, Erato Stylianou-Markidou, Ioannis Pantalos, Constantinos Koumenis, Alexandros G. Georgakilas

**Affiliations:** 1 DNA Damage Laboratory, Physics Department, School of Applied Mathematical and Physical Sciences, National Technical University of Athens (NTUA), Zografou Campus, 15780 Athens, Greece; spkals@central.ntua.gr (S.A.K.); angelikigkikoudi@mail.ntua.gr (A.G.); or a.koutsostathis@kit.edu (A.K.); adamopoulousissy18@gmail.com (A.A.); svasilopoulos@mail.ntua.gr (S.N.V.); ipantalos@me.com (I.P.); costas.koumenis@pennmedicine.upenn.edu (C.K.); 2 Karlsruhe Institute of Technology (KIT), 76344 Eggestein-Leopoldshafen, Germany; 3 School of Medicine, National Kapodistrian University of Athens (NKUA), 11527 Athens, Greece; rangosvassilis@gmail.com; 4 University of Cyprus, 1 Panepistimiou Avenue, 2109 Aglantzia, Nicosia P.O. Box 20537, Cyprus; stylianou-markidou.erato@ucy.ac.cy; 5 International Radiotherapy Services Ltd., Larnaka 373022, Cyprus; 6 Department of Radiation Oncology, Perelman School of Medicine, University of Pennsylvania, Philadelphia, PA 19104, USA

**Keywords:** proton therapy, relative biological effectiveness, linear energy transfer, spread-out Bragg peak, complex DNA damage, radiosensitivity, radioresistance, clustered lesions, distal-edge effect, immunotherapy synergy

## Abstract

Background/Objectives: Proton therapy has emerged as an advanced radiotherapy modality due to its unique physical dose distribution and its distinct radiobiological properties. The finite range of protons in tissue enables highly conformal dose delivery with minimal exit dose, significantly reducing irradiation of surrounding normal tissues compared to photon-based radiotherapy. Beyond these physical advantages, proton beams exhibit a spatially varying linear energy transfer that increases toward the distal edge of the spread-out Bragg peak, leading to clustered and complex DNA damage that is more difficult for cancer cells to repair. Methods: This review integrates experimental, computational, and clinical evidence to examine how proton-induced DNA damage, relative biological effectiveness, oxygen effects, and non-targeted responses contribute to tumor control and normal tissue sparing. Results: Comparative analyses with photon intensity-modulated radiotherapy demonstrate consistent reductions in acute and late toxicities across multiple tumor sites, particularly in pediatric patients and in tumors located near critical organs. The review also discusses emerging technologies, including pencil beam scanning, image-guided and adaptive proton therapy, compact accelerator systems, and ultra-high dose rate FLASH proton therapy, which collectively aim to enhance treatment precision, biological effectiveness, and accessibility. Conclusions: Together, these developments support proton therapy as a rapidly evolving modality with significant potential to improve therapeutic outcomes in modern oncology.

## Simple Summary

A key advantage of protons is their ability to stop at a defined depth inside the body, allowing high radiation doses to be delivered to tumors while reducing damage to surrounding healthy tissues. In addition to these physical benefits, protons produce more complex damage within cancer cells, particularly near the end of their path, which can make tumors harder to repair and more sensitive to treatment. This review summarizes current knowledge on the physical principles, biological effects, and clinical outcomes of proton therapy and compares it with modern photon-based techniques. Emerging developments, including advanced beam delivery, integration with imaging and artificial intelligence, and ultra-high dose rate (FLASH) proton therapy, highlight the growing role of proton therapy as a precise, biologically effective, and evolving modality in cancer care.

## 1. Introduction

**Figure 1:**
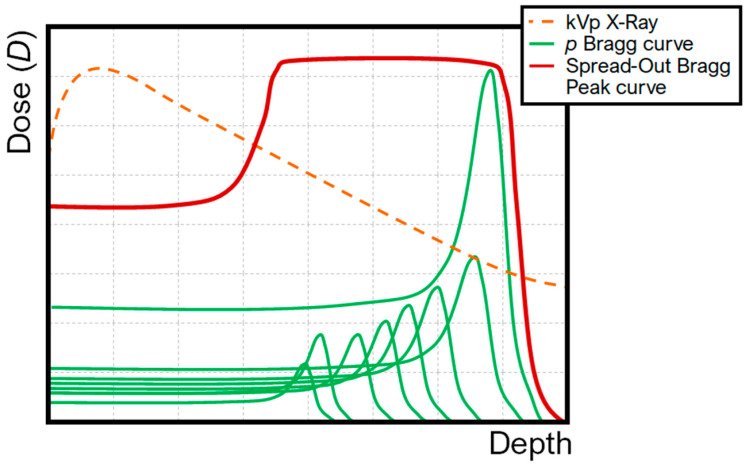
Schematic of the superposition of multiple proton Bragg curves into a single SOBP and qualitative comparison with the longitudinal dose profile of kVp X-rays.

Proton therapy exploits the finite range of protons in tissue to deliver highly conformal radiation doses to tumors while minimizing exposure of surrounding healthy structures. Accelerated protons deposit minimal energy along their entrance path and release the majority at a defined depth—the Bragg peak—determined by their initial energy. Beyond this peak, dose falls to near zero within millimetres, eliminating the exit dose characteristic of photon radiotherapy [1,2]. Energy loss occurs primarily through inelastic Coulomb interactions with atomic electrons, governed by the Bethe–Bloch equation [3]. The rate of energy loss per unit path lengthincreases inversely with the square of proton velocity, resulting in the characteristic Bragg curve: a low-dose entrance plateau, a sharp peak, and an abrupt distal fall-off (Figure 1). To cover clinically relevant target volumes, multiple pristine peaks of varying energy are superimposed to form a spread-out Bragg peak (SOBP) using passive scattering or pencil-beam scanning [4]. Carbon-ion therapy extends these principles, with higher-charge particles providing even greater biological effectiveness [5].

Over the past decade, the rapid global expansion of proton therapy facilities, together with advances in beam delivery, Monte Carlo modeling, and molecular radiobiology, has renewed interest in understanding how these biological effects translate into clinical benefit. At the same time, important questions remain regarding variable relative biological effectiveness (RBE), LET-based treatment planning, oxygen effects, and non-targeted biological responses (biological effects observed in cells or tissues that are not directly traversed by radiation tracks but arise due to signaling from irradiated cells, including radiation-induced bystander and abscopal effects), all of which challenge the traditional assumption of a constant RBE value of 1.1 used in clinical practice. In this review, we provide an integrated synthesis of the physical principles, radiobiological mechanisms, and emerging clinical evidence underlying proton therapy. We focus on proton-induced DNA damage complexity, LET-dependent biological effects, comparative outcomes with modern photon radiotherapy, and recent technological developments, including image-guided delivery, adaptive proton therapy, and ultra-high dose rate FLASH proton therapy. By bridging physics, biology, and clinical practice, this review aims to clarify the current state of proton therapy and highlight future directions for biologically informed and precision-based radiation oncology.

## 2. Radiobiological Mechanisms of Proton Therapy

At the cellular level, the interactions of protons with biological matter induce a variety of biological damage in cells and tissues. Protons deposit energy in tissue mainly via Coulomb interactions, where the positively charged protons interact with the electrons and nuclei of atoms in the cell. These interactions cause excitation and ionization of atoms and molecules along the proton’s path. The rate of energy deposition is described as linear energy transfer (LET) [1]. One of the critical aspects of proton-induced DNA damage is the clustering of lesions near the Bragg peak. As protons approach the end of their path, they induce densely ionizing tracks, creating regions of highly complex DNA damage (see also Table 1). DNA damage is strongly connected to DNA mutations, cell death and ultimately cancer. Protons may induce a plethora of DNA damage, from base and sugar damage to DNA–protein and DNA–DNA cross-links and from single-strand breaks (SSBs) to complex double-strand breaks (DSBs) and non-DSBs. Clustered DNA damage is more biologically significant than isolated single lesions because the relevant repair mechanisms, i.e., non-homologous end joining (NHEJ) and homologous recombination (HR), are less efficient at dealing with complex, closely spaced damage. This makes proton-induced DNA damage more cytotoxic, particularly at the Bragg peak, where LET is highest. The high LET of protons in the Bragg peak region results in densely clustered DNA damage that is more difficult for cells to repair in comparison to damage caused by low-LET radiation, like X-rays. This densely ionizing radiation (IR) increases the likelihood of cell death in cancerous tissues, enhancing the treatment’s effectiveness. Belli et al. investigated the inactivation effects of low-energy protons on human normal and tumor cells, showing that the RBE increases with LET. In that study, they measured incident LET values ranging from 7.6 to 29.6 keV μm^−1^ and demonstrated that radioresistant tumor cells, such as SQ20B, showed the highest increase in proton effectiveness at higher LET, with an RBE (2 Gy) of up to 3.2, while normal cells like HF19 showed minimal variation, emphasizing the differential sensitivity of tumor cells to high-LET protons [6].

The long-term study on proton therapy for localized prostate cancer demonstrated that high-dose proton therapy, while achieving excellent disease control, is associated with a low incidence of severe genitourinary and gastrointestinal toxicities. However, the findings suggest that reducing the total dose from 78 Gy to 76 Gy may minimize late toxicities without compromising biochemical control, highlighting the importance of dose optimization in proton therapy [7]. While these results support the safety and efficacy of proton therapy, current evidence does not provide definitive proof of clinical superiority over modern photon-based radiotherapy. Direct comparative trials have not consistently demonstrated a clear advantage in clinical outcomes, and therefore conclusions regarding superiority should be drawn with caution. The potential radiobiological advantages of particle therapy associated with increased LET are more relevant in the treatment of radioresistant tumors, such as sarcomas [8], for which conventional photon radiotherapy often yields limited local control. In these contexts, high-LET radiation may offer improved biological effectiveness compared with X-ray therapy.

### Illustrative Monte Carlo Examples of LET-Dependent DNA Damage Based on Published Models

This subsection presents illustrative Monte Carlo examples based entirely on previously published and validated track-structure models. The purpose is purely didactic: to visualize well-established LET-dependent trends in DNA damage complexity. No new biological mechanisms, quantitative predictions, or clinically actionable conclusions are derived from these examples.

Monte Carlo track-structure simulations have played a central role in elucidating how proton radiation quality translates into biological damage at nanometric scales. Increasing proton LET leads to progressively denser ionization patterns along particle tracks, resulting in highly clustered DNA damage and complex DSB configurations, particularly at low proton energies. These simulations have provided quantitative evidence that proton-induced DNA damage complexity increases with decreasing proton energy, supporting the concept of variable biological effectiveness along the proton track. To illustrate LET effects on DNA damage complexity, we adopt the Monte Carlo DNA damage simulation (MCDS) framework as described by Nikitaki et al. [9]. The MCDS code has been extensively validated and applied to estimate radiation-induced DNA damage yields as a function of particle type, energy, and oxygenation conditions. Representative parameter sets previously reported [9] are shown here as illustrative examples for proton irradiation under normoxic (20% O_2_) and hypoxic (0.1% O_2_) conditions at two characteristic regions of the proton track: the beam entrance (250 MeV) and near the Bragg peak (100 keV).

These illustrative examples reflect the well-established LET-dependent increase in clustered DNA damage near the Bragg peak (Table 2 and Table 3). Such LET-dependent increases in damage complexity provide a biological rationale for ongoing efforts to incorporate LET and variable RBE considerations into proton therapy treatment planning.

In Table 4, the fraction of complex damage among DSBs shows that, at the entrance of the beam, about half of the lesions in clusters are non-DSBs, which is often seen in low-LET radiation and suggests that most damage can be more easily repaired by the corresponding cell mechanisms. On the other hand, at the Bragg peak region, most of the damage is due to DSBs, which are generally harder to repair. In all cases, for low-energy protons, the influence of oxygen is less significant compared to regions with high energy and, subsequently, low-LET radiation.

These illustrative examples highlight the physical and biological basis of the Bragg peak phenomenon. Further in vivo research is necessary to evaluate the advantages of proton therapy in living organisms. It is worth mentioning that these representative simulations are presented to illustrate well-established trends in LET-dependent DNA damage complexity along the proton track, rather than to provide an exhaustive or patient-specific modelling study.

## 3. DNA Damage and Non-Targeted Effects of Proton Irradiation

Proton irradiation primarily targets DNA through both direct energy deposition and indirect effects mediated by reactive species generated during water radiolysis. In aerobic conditions, water radiolysis produces reactive oxygen species (ROS), including hydroxyl radicals, peroxyl radicals, and hydroperoxides, which are highly damaging to DNA. In addition, reactive nitrogen species (RNS), such as peroxynitrite formed from nitric oxide and superoxide radicals, contribute to oxidative damage [10,11]. These reactive species activate intracellular signaling pathways that enhance oxidase expression, leading to increased oxidative stress that may exceed the cell’s antioxidant defense capacity [12,13]. Various techniques have been developed over recent decades for the measurement and in vitro quantification of such lesions induced after proton irradiation of cells (e.g., immunostaining for the detection of repair proteins, such as γ-H2AX and 53BP1) [14,15,16].

As mentioned above, it has been shown that even the passage of a single particle through biological matter may induce such complicated lesions at low doses [17]. The complexity of mutations due to clustered lesions is the result of different degrees of gross chromosomal rearrangements (inversions, translocations, deletions, partial and even complete loss of chromosomes) and, when induced in human somatic cells, especially combined with the activation of proto-oncogenes or the inactivation of tumor suppressors, it may lead to carcinogenesis [18]. In this way, different types of induced chromosome aberrations, such as two chromosome breaks on two distinct chromosomes (which may lead to a ‘dicentric’ or a ‘reciprocal translocation’), or in the same chromosome (which can generate a ‘ring’), and a single un-rejoined chromosome break (which may lead to a ‘terminal deletion’), as well as gross chromosome aberrations (i.e., those involving three or more breaks in two or more chromosomes [19]) depend on the target characteristics (the DNA organization in the cell nucleus, i.e., the percentage of euchromatin, which is more accessible and thus more prone to damage, and the phase of the cell cycle) as well as the part of the genetic molecule affected by the particle irradiation [20].

Furthermore, another fact about proton irradiation is that unirradiated cells can respond when neighbouring cells are irradiated, a phenomenon known as the radiation-induced bystander effect. Such effects in neighbouring nontargeted cells include DNA damage, mutations, chromosomal instability, an increased possibility of apoptosis, necrosis, micronucleation, affected activity of regulatory proteins and relevant enzymes, senescence, inflammation, proliferation and oncogenic transformation [21,22,23]. These effects are attributed to signals released by the irradiated cells—mainly due to the induction of ROS and RNS—followed by the activation of stress-sensitive kinases ATM, p53, MAPK and transcription factors AP-1 and NF-kB and secondary induction of COX-2, iNOS, prostaglandins, pro-inflammatory cytokines, growth factors and chemokines [24,25,26]. Although the bystander effect is considered a highly complicated process due to a plethora of interfering factors among neighbouring cells—the irradiated and the bystander ones—the latter respond differently in normal and cancer cells. For example, an absorbed dose of 5.1 Gy of proton microbeam irradiation of lung cancer cells induces DSBs in bystander cancer cells but not in normal lung fibroblasts, while proton irradiation with a dose of 3.8 Gy of lung fibroblasts does not induce bystander effects in lung cancer cells [27]. At low-dose proton irradiation, the effects in targeted and bystander cells are nearly the same [28]. The range of horizontally transmitted bystander signals is also considered high, since studies have recorded the involvement of 10–20 cells in bystander effects around the targeted point [29,30].

Local tumor recurrence and second cancer development are other fundamental aspects of PT targeting at the tissue level. Safe conclusions on tumor recurrence after PT are difficult to draw, since contemporary radiation treatment planning depends on various variables that confound comparisons between different radiation therapies at the clinical level. A cohort study by Xiang et al. (2020) showed that the relative risk of a second primary cancer is lower after PT than after modern photon RT [31]. Further controlled studies in this field are necessary to elucidate whether the risk of local cancer recurrence and/or second cancer development following PT is lower than that following conventional photon RT. Beyond local DNA damage mechanisms, proton therapy may also exert favorable systemic biological effects. Preclinical studies have demonstrated that proton irradiation induces significantly less radiation-induced lymphopenia compared with photon irradiation, reflecting improved sparing of circulating immune cells. This immune-preserving effect may be particularly relevant for tumors treated with large fields or in combination with immunotherapeutic approaches, highlighting an additional biological advantage of proton therapy beyond physical dose conformity [32].

## 4. Proton Versus Photon Radiotherapy: Dosimetric, Biological and Clinical Comparisons

Radiation therapy is an integral component of cancer treatment worldwide and is commonly used in the management of cancer patients. Photon therapy is the most common type of RT because of its long-term utilization since the very early stages of cancer treatment, its availability, and its low-cost access. Its efficacy and outcomes have been studied in depth, and it is considered the basis of nearly every cancer treatment. On the other hand, proton therapy, which has been in use since 1952 [33], is a promising type of therapy with explosive growth over the last two decades, regarded as a method of choice for many types of cancer. This is especially due to its dose distribution not only on a macroscopic scale but also at the microscopic scale of radiation track structure and localized energy deposition, which results in a reduced dose to the normal tissues surrounding the tumor. It is of note that PT was applied at its first stages of development for the treatment of radio-resistant tumors and during the last three decades has been expanded to irradiate other types of cancer such as pancreatic, prostate, breast, hepatocellular, head and neck, as well as pediatric cancers. A high-level comparison of proton therapy and photon intensity-modulated proton therapy (IMRT) across key clinical and dosimetric features is summarized in Table 5.

The radiobiological effects of photons, and protons seem to be similar and therapeutic proton beams are considered low-LET radiation. While the main target in RT is focused on eliminating the tumor while minimizing the irradiation of the surrounding healthy tissue, the advances in proton RT are the increase in the therapeutic ratio (i.e., the difference between tumor control and normal tissue toxicity) [34]. Because of the Bragg peak, proton beams deposit most of their energy in the tumor while minimizing exit dose, unlike photons which deliver dose along their entire path. In the case of protons, the thin peak due to a single proton is broadened into the Bragg peak by the protons of the beam, delivering a homogeneous dose in the tumor but still needing multiple beam angles for the optimization of the dose. While protons slow down with increasing length in biological matter, they are also scattered laterally, producing a penumbra that surrounds the boundary of the beam. Photon-based radiotherapy may translate into reduced acute and late toxicities in selected clinical scenarios, particularly for tumors located near critical organs. In head and neck cancer, a recent prospective study using objective biophysical skin measurements reported comparable acute and early-late cutaneous toxicity between modern photon (VMAT) and proton (IMPT) radiotherapy, with superficial skin dose and patient phenotype emerging as stronger predictors of skin toxicity than treatment modality itself [35]. These findings highlight that toxicity outcomes in proton therapy are highly site- and dose-dependent and underscore the importance of indication-specific evaluation rather than assuming uniform toxicity reduction across all clinical settings.

Protons sustain an optimized fundamental in RT by delivering higher doses to the target tumor while maintaining the dose delivered to the surrounding tissues. Generally, the total dose delivered to the patient’s body is lower in the case of proton RT. The present PT, even compared to the latest photon irradiation techniques, such as IMRT and volumetric modulated arc therapy (VMAT), may still deposit a reduced whole-body radiation dose by 50–60% for similar doses to the target tumor [36]. On the other hand, comparing the dose conformity between proton and photon RT, it has been proven that photons excel, especially after the development of intensity-modulated photon therapy [37]. Proton RT shows great advantages for tumors located close to critical anatomical structures and sensitive tissues, where a small overdose may cause serious complications, e.g., in the case of skull base chordoma [38], as well as nasopharyngeal carcinoma and intracranial tumors [39]. Proton therapy has achieved dosimetric protection of radiation-sensitive anatomical regions such as the thyroid gland, breast, prostate, etc. The same dosimetric advantages of PT are applied to the irradiation of pediatric patients, where the total absorbed energy is decreased by a factor of 2–3 in comparison to the photon irradiation [40]. Thus, protons have produced better toxicological profiles compared to photon RT.

There is substantial evidence indicating that proton therapy is particularly advantageous compared with photon radiotherapy in the setting of re-irradiation following tumor recurrence. When full-dose re-irradiation is required, photon-based treatments are often limited by cumulative dose to surrounding normal tissues, resulting in reduced local control. In contrast, proton therapy enables improved dose conformity and normal tissue sparing, leading to higher achievable tumor doses and improved local control rates [41,42]. Unlike photon irradiation systems, which offer limited further improvement in dose distribution due to unavoidable exit dose, proton therapy provides enhanced dose-sculpting capabilities through techniques such as IMPT. These capabilities allow radiation dose to be more precisely confined to the tumor volume and its immediate margins, thereby reducing exposure of adjacent healthy tissues.

Importantly, while the most significant disadvantage of proton therapy—in comparison to photon therapy—until now has been thought to be its cost, several studies have shown a different perspective on the total cost-effectiveness balance between the two therapies. Since 2005, Lundkvist et al. have reported superior cost-effectiveness of proton therapy for medulloblastoma patients using Markov modeling, taking into account the corresponding costs of adverse effects [43]. Similar results were also recorded in other studies [44,45,46]. It is true that proton therapy involves substantially higher upfront infrastructure costs than conventional photon therapies when only the initial treatment cost is considered. However, unlike photon linear accelerators, which typically represent stand-alone treatment units, a single proton accelerator is commonly designed to serve multiple treatment rooms within the same facility. This design distributes capital investment across several gantries and alters per-room and per-patient cost considerations.

The overall cost of proton therapy is therefore closely linked to the construction and operational scale of the proton treatment center, as well as to the proportion of patients who may appropriately benefit from proton therapy, which in turn depends on national healthcare systems and economic factors. In parallel, proton therapy is increasingly adopted worldwide due to its favorable dosimetric characteristics, and continued technological development is expected to contribute to cost reductions over time. This trend may support more individualized patient selection strategies, improving the overall cost-effectiveness of proton therapy [47].

## 5. Comparison Between Proton Radiation and Other Charged Particle Therapies

Hadron therapy, as a branch of RT, has the benefit of precise dose localization, and, as noted above with protons, it is based on the fact that particle beams show a steep and localized dose peak (Bragg peak), which is used to deposit a precise radiation dose to the tumor with reduced dose deposition to the neighbouring normal tissues. The dosimetric superiority of charged particle beams therefore led to their utilization in modern RT worldwide, with protons adopted as the leading hadron therapy modality in the US and Europe, while carbon ion RT was developed and further clinically studied in Germany and Japan. For example, it has been shown that proton RT reduces the likelihood of severe radiation-induced lymphopenia in cancer patients [48]. Proton therapy reduces the likelihood of high-grade radiation-induced lymphopenia in glioblastoma patients, as demonstrated in a phase II randomized study of protons vs. photons [49,50].

One of the basic characteristics of solid tumors is hypoxia, which is associated with radioresistance, an increased possibility of distant metastases and, subsequently, poor prognosis in cancer patients. The oxygen enhancement ratio (OER), i.e., the ratio of absorbed doses required to produce the same biological effect for irradiations in the presence and absence of oxygen, takes values in the range of 2.5–3.0 for low-LET radiations, such as conventional photons (*γ*- and X-rays). The OER asymptotically approaches 1.0 for high-LET irradiations [51]. Hadron RT also has the potential to reduce secondary cancers. According to a Japanese study comparing the risk of secondary malignancy between prostate cancer patients treated with carbon ion therapy and those treated with photon RT, carbon ion RT was associated with a lower corresponding risk [52]. In a similar Chinese study of 2020, based on the post-treatment results of 450,000 patients—according to the Chinese National Cancer Database—who had received three-dimensional conformal RT, IMRT and proton beam RT, the latter was associated with a lower risk of second cancer [31]. Related findings have been documented for helium ion RT [53,54].

Carbon ions make use of the characteristic Bragg peak to improve dose–depth distribution, being heavier than protons (with a higher LET value than that of protons) but with similar physical properties. Carbon ions were first utilized for RT in Chiba, Japan, in 1994 (at the renamed National Institute for Quantum and Radiological Science and Technology) [55], and subsequently in other European countries [56]. Their therapeutic beams have LET values in the range of 10–80 keV μm^−1^, while the corresponding proton beams range between 0.4 and 30 keV μm^−1^ [57]. Carbon-12 ions show reduced longitudinal and lateral scattering compared to protons, leading to a smaller dose halo, but they deliver densely ionizing radiation in the Bragg peak region [58]. They also have a fragmentation tail beyond the Bragg peak, which is attributed to nuclear interactions with the atoms of the irradiated healthy tissue. These ions show high RBE values, with the biological RBE at approximately 1.5 and the corresponding clinical RBE at 3.0 [59]. Being high-LET ions, they exhibit a reduced dependence on fractionation and the cell-cycle stage of the irradiated cells. In addition, they show a reduced oxygen enhancement ratio (OER), which is advantageous for the effective killing of hypoxic and generally radioresistant cancer cells. These high RBE and low OER values make them well suited for cancer radiotherapy. Different radiation modalities may also differentially affect responses to chemoradiotherapy (CRT) and PD-L1 expression in tumor cells under normoxic and hypoxic conditions. In this context, further clinical trials are required to provide more definitive evidence regarding the effectiveness of carbon ion radiotherapy in controlling hypoxic, radioresistant tumors. Existing evidence shows that carbon ions are more effective in treating head and neck cancers due to the fact that they provide a narrow irradiating volume compared with protons [60]. Clinical results of carbon-ion radiotherapy have demonstrated favourable outcomes compared with historically reported results for conventional photon and proton radiotherapy [61]. In patients with radioresistant tumors, high local control and survival rates have been observed, supporting the potential clinical advantage of carbon ions in selected clinical indications [62]. In a recently published meta-analysis of the efficacy and safety of proton and carbon ion radiotherapies [63], the authors drew the conclusion that these RTs show comparable oncological outcomes and toxicities, and, therefore, more research on this topic is needed in the future. Carbon ion RT still has a significant disadvantage, which is its cost; it is estimated that the installation cost for this kind of RT is approximately EUR 140 million, compared to EUR 95 million for the corresponding proton facility and EUR 23.4 million for a photon facility. For carbon beams, there is also a treatment cost of about EUR 1128 per fraction, while the costs for protons and photons are EUR 743 and EUR 233, respectively [64].

Helium ion (4He) beam therapy, which has been used clinically since 1975 at the Lawrence Berkeley National Laboratory [65], has not progressed over the years; it has remained in an elementary application state, clinically unexploited, although helium ions have intermediate physical and biophysical properties between protons and carbon ions. More specifically, compared to protons, helium ions show a sharper Bragg peak, reduced lateral scattering, a sharp lateral penumbra, range straggling with a higher RBE value (~1.3–3.0), enhanced LET and a decreased fragmentation tail for the higher energies used clinically [66]. Helium ions can achieve higher LET values than protons and therefore provide a more favorable alignment of the Bragg peak with therapeutically relevant high-LET regions. In parallel, for helium ions, the RBE value (its range lies between 1.3 and 3.0) increases as a function of LET, and, therefore, they may deliver high doses to the target tumor while minimizing low doses to the surrounding normal tissue. Currently, there are only a few helium beam therapy facilities (in Europe, China and Japan), with the most promising being the Heidelberg Ion-Beam Therapy Center in Germany, which has had a helium ion source installation since 2015, using raster-scanning technology [67], already in clinical use [68].

## 6. Technical Aspects of Proton Therapy

The first application of brachytherapy took place in 1901, implementing a ^226^Ra α-particle source [69]. The first human patients who received proton radiation were treated at Lawrence Berkeley Laboratory; they suffered from breast cancer and were treated by irradiation of the pituitary gland in order to induce hormone suppression [33]. Numerous experimental installations, typically hosted at nuclear physics research facilities, provided optimistic results for the clinical application of proton therapy [70]. The first hospital-based proton irradiation facility commenced operation in 1990 at the Loma Linda University Medical Centre in California [71]. Numerous clinical facilities have operated ever since, and, as of December 2024, there are 121 centres for proton RT around the world [72].

### 6.1. Main Characteristics of Cyclotron and Synchrotron Facilities

**Figure 2:**
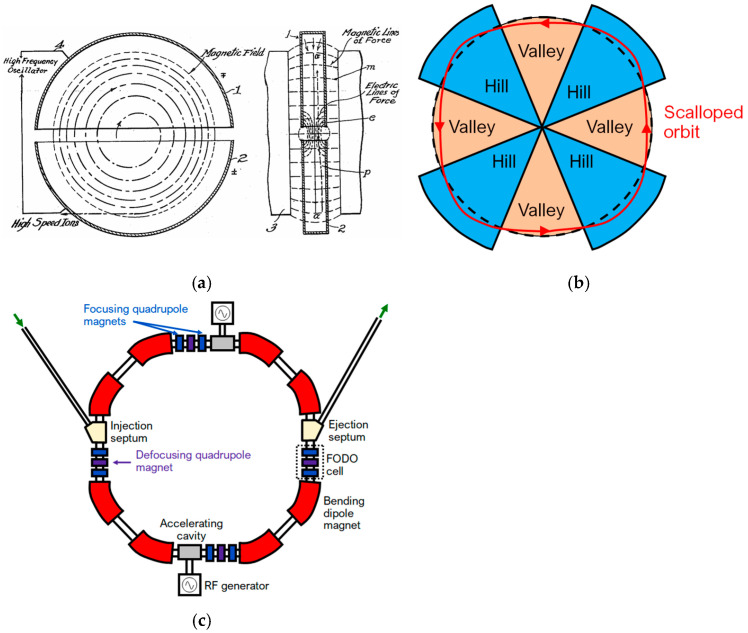
Main concepts of cyclotron and synchrotron. (**a**) The concept of the cyclotron from E. O. Lawrence’s patent (US Patent 1948384) [74,75]; (**b**) Dees’ structure and particle orbit of an AVF cyclotron; and (**c**) Essential elements of a synchrotron (only a few of the repetitive elements are shown).

The cyclotron was originally proposed by Ernest O. Lawrence in 1930 and has seen remarkable developments since that year [73]. The first cyclotrons were able to accelerate protons and deuterons up to several tens of mega-electronvolts, while modern cyclotrons can produce beams of greater power than those in the 1930s and can accelerate ions as heavy as uranium [74]. Modern particle therapy cyclotrons accelerate protons to a fixed energy of up to 250 MeV. The proton beam is produced at the centre of the cyclotron by a hydrogen-ion source. The acceleration is conducted by the radiofrequency (RF) electrodes (D’s, or Dees), and the beam is bent by a strong magnet. The protons are then extracted from the cyclotron’s magnetic field into a beam transport system, which allows for further energy and range modulation. The acceleration of the particles is facilitated by the electric field in the gap between the Dees, and repeated revolutions of the protons inside the cyclotron allow for high-energy beams [2]. A schematic of the main elements of a typical classical cyclotron setup is shown in Figure 2a. One main disadvantage of the aforementioned classical cyclotrons is that, due to relativistic effects, the process requires significantly higher voltage to further accelerate highly energetic particles, as the revolution frequency decreases with energy. The result of this issue is that classical cyclotrons can typically accelerate protons only up to about 20 MeV. There are two cyclotron models that can bypass these limitations: the synchrocyclotron and the AVF (Azimuthally Varying-Field) cyclotron. Synchrocyclotrons facilitate the production of beams of relativistic velocity by reducing the RF of the accelerating voltage, thus allowing the revolving particles to synchronise with the RF voltage and reach higher energies. However, the repetition cycle of this RF modulation generates a gap between the extracted beam from the ion source and the accelerated beam, which significantly limits the beam intensity by two to three orders of magnitude compared to the fixed-RF cyclotrons [73]. The most common accelerator used in modern RT facilities is the AVF cyclotron, also frequently referred to as an isochronous cyclotron. Its main characteristic is the alternation of the magnetic field of the Dees, causing a scalloped orbit, as shown in Figure 2b. The transition between areas of high (hills) and low (valleys) magnetic field has a focusing effect on the beam, and the proportionality of the mean magnetic field to the particle’s energy allows for the maintenance of isochronism [75].

The first synchrotron was proposed by Edwin M. McMillan in 1945 [76]. The synchrotron comprises a series of acceleration cavities, bending magnets and focusing elements, as shown in Figure 2c. Particles are injected into the ring, where they are then accelerated by several radiofrequency cavities, and their trajectory is bent by a set of bending dipole magnets. Quadrupole and sextupole magnets are also used to enhance the characteristics of the beam, as they function as focusing elements for its spatial and energy distribution, respectively [77]. The acceleration process in a synchrotron takes place in cycles (spills); each spill consists of filling the ring with a bunch of protons. The latter are accelerated until the desired energy is reached and then extracted, followed by the deceleration and dumping of unused particles. Synchrotron beams are highly sensitive to deviations in the acceleration line parameters, such as alignment errors, due to the large number of protons in the beam (on the order of 10^9^) [2].

### 6.2. Dose Delivery Techniques

**Figure 3:**
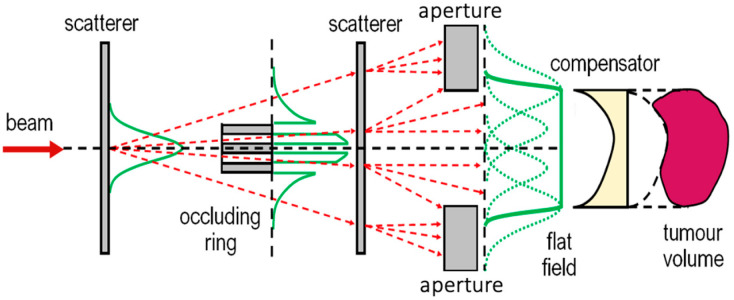
Essential beamline elements and their interaction with the beam for the formation of a doubly scattered flat field, featuring an occluding ring and a compensator. Dashed red lines indicate components of the scattered beam, and green lines indicate the lateral beam profile with and without (dashed) aperture.

One of the significant advantages of hadron therapy is the enhanced selectivity offered by the Bragg peak. However, the effect of the Bragg peak is not enough to offer optimal selectivity, and numerous passive and dynamic techniques for enhanced delivery have been developed. Furthermore, the beam, which is extracted from the accelerator, is monoenergetic and has an emittance of a few millimetres [2]. Modern dynamic techniques implement the Pencil Beam Scanning (PBS) technique, while passive techniques focus on the shaping of the beam that reaches the irradiation field and rapidly modulate its energy, allowing the formation of an SOBP that matches the tumor volume. The systems that implement these techniques constitute what is widely referred to as the nozzle. Energy modulation can also take place upstream, with the implementation of a degrader followed by an Energy Selection System (ESS) [78]. Passive shaping of the beam is conducted via the use of scattering and collimating materials (Figure 3). Scatterers, made of materials of high atomic number (*Z*), such as lead or tantalum, in combination with collimators, provide a shape-specific uniform irradiation field. At the irradiation field, a range compensator, typically made from low-*Z* materials (e.g., plastic), is placed to shape the irradiation field according to the tumor volume and any potential abnormalities of the preceding tissue. A range modulator wheel consists of a propeller-shaped wheel, made from low-*Z* materials, with each blade being of a different thickness. The addition of this element of the nozzle allows the longitudinal spreading of the Bragg peak over the tumor volume and results in the formation of an SOBP. There are numerous versions of the scattering method: single-foil scattering, double-foil scattering, and double scattering with a dual or an occluding ring. One of the issues with passive scattering techniques is the generation of secondary neutrons, which require shielding at strategic locations [79,80].

**Figure 4:**
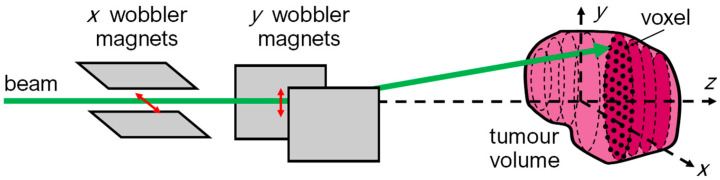
Beam deflection by the wobbler magnets for implementation of the PBS technique.

In PBS, the tumor volume is divided into voxels and the scanning system is programmed to place the Bragg peak inside each voxel. The scanning of the volume encompasses the lateral scanning of the beam by a set of orthogonal dipole (wobbler) magnets, as shown in Figure 4. Scanning in the beam direction (*z* axis, in Figure 3) is conducted via the energy modulation of the beam, either at accelerator level (upstream modulation) or via a range shifter. This method allows for the irradiation of fields of all shapes and sizes; however, it is highly sensitive to organ motion (Figure 4). Recent work has shown that uncertainties in deformable image registration can significantly affect dose accumulation reliability in adaptive proton therapy, highlighting the importance of accounting for accumulated dose uncertainty already at the treatment planning stage [81].

## 7. FLASH Radiation Therapy

Ongoing research has demonstrated that increasing the dose rate of RT from conventional levels (typically less than 0.1 Gy s^−1^) to Ultra-High Dose Rates (UHDR) exceeding 40 Gy s^−1^ (FLASH-RT) can significantly enhance the sparing of normal tissues [82]. This is called the “FLASH Effect” and has shown great potential to revolutionize RT. Unlike conventional RT, which typically administers a therapeutic dose within several minutes over multiple sessions, FLASH treatment delivers the entire radiation dose in less than a second. Proton and electron beams are currently used for this purpose. Generating X-rays at FLASH dose rates is much more difficult because those photons are produced by bremsstrahlung radiation, where the conversion efficiency is very low. Manufacturers aim to create commercially viable clinical accelerators capable of delivering high-charge-density bunches to achieve FLASH dose rates. The reason why normal cells are spared when irradiated with UHDR radiation is not well known. Many hypotheses have been proposed, including the following:

(a) Radiolytic Oxygen Depletion

Studies have shown that when a tissue is irradiated with UHDR radiation, the oxygen levels are rapidly reduced, causing the irradiated cells to temporarily become hypoxic and more radioresistant. Other studies in which oxygen levels were directly measured concluded that no total oxygen depletion was observed. Thus, this hypothesis alone is not adequate to explain the FLASH effect. Instead, a reduction in oxygen consumption has been observed at higher dose rates, which is associated with lower steady-state levels of electron radicals [83].

(b) The Role of Reactive Oxygen Species (ROS)

When cells are exposed to FLASH radiation, fewer free radicals are produced. Cells are consequently less sensitive to radiation. In conventional RT, lipids contribute to the production of ROS, but in the case of FLASH, the dose is delivered quickly, reducing lipid peroxidation and sustaining normal cells. Fenton chemistry also plays an important role, as normal tissues can regulate the amount of labile (readily available) Fe in comparison to cancerous cells, thus reducing the damage in normal cells. In addition, normal tissues have mechanisms (enzymatic) that remove ROS more efficiently than tumors. It has also been proposed that free radicals could recombine, resulting in better tissue sparing [84].

(c) Immune sparing hypothesis

Lymphocytes and other circulating blood cells are exposed for a very short amount of time (<1 s) to FLASH in comparison to conventional methods in which the organism is exposed for a few minutes. As a result, a smaller percentage of healthy circulating cells are preserved, potentially reducing the odds of common side effects related to RT ion therapy, such as lymphopenia [85].

This differential activation between healthy and tumor tissues may contribute to FLASH’s ability to spare normal tissue while maintaining tumor control. Studies performed with proton FLASH are fewer than those with electron/photon irradiation and are generally compared with conventional photon therapy as a control [86]. Currently, research trends have expanded towards FLASH-RT with proton and electron beams. While most studies have focused on electron FLASH-RT (eFLASH-RT), which has the disadvantage of being unsuitable for irradiation of deep-seated tumors, there is no lack of promising results from proton FLASH-RT (pFLASH-RT). The first clinical trial, FAST-01, consisted of the treatment of bone metastases in the extremities, incorporating the Varian ProBeam system at Cincinnati Children’s/UC Health Proton Therapy Centre; it was facilitated with a monofractional 8 Gy dose delivery at a dose delivery rate of 51–61 Gy s^−1^, implementing the open-field-transmission and PBS methods [87]. Furthermore, the FLASH laboratory of the Perelman School of Medicine at the University of Pennsylvania has modified the transmission line of an IBA Proteus Plus cyclotron, incorporating passive double-scattering alongside CT-defined geometry of the irradiation field. The aforementioned setup is used in preclinical experiments [88].

## 8. Conclusions and Future Perspectives

**Figure 5:**
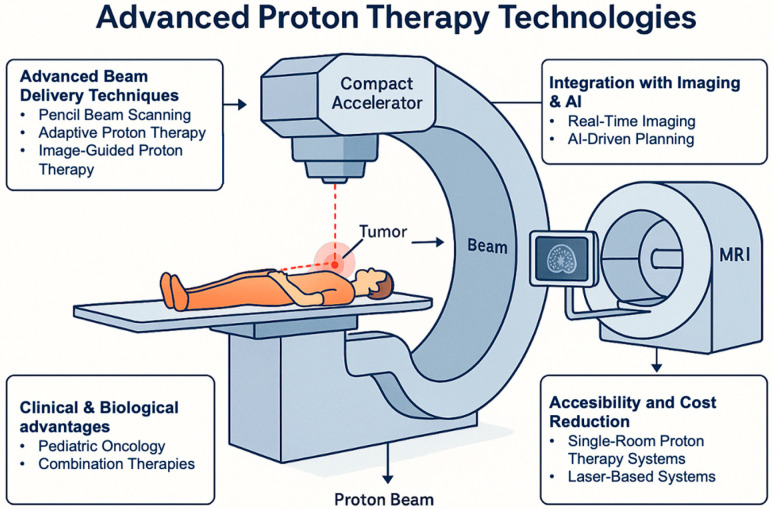
Schematic representation of the gantry head, illustrating beam delivery and modulation components (e.g., scanning magnets, monitoring systems, and range modulation devices). Drawing created using Adobe Illustrator. (www.adobe.com, accessed on 5 January 2026).

Proton therapy represents a technologically advanced radiotherapy modality that combines highly conformal physical dose distribution with distinct radiobiological characteristics. The depth–dose profile defined by the Bragg peak enables substantial reduction of exit dose and improved sparing of surrounding normal tissues, particularly in an-atomically complex regions and in pediatric patients. In parallel, the spatial variation in linear energy transfer along the proton track contributes to increased DNA damage complexity near the distal edge, raising important questions regarding variable relative bio-logical effectiveness and its clinical implications. In light of the historical dosimetric and normal-tissue-sparing advantages of proton therapy outlined in earlier clinical reviews, and reinforced by emerging high-level data showing significant improvements in survival and reduced treatment-related toxicity in randomized trials, proton therapy stands poised to transition from a theoretically superior modality to one with demonstrable clinical benefit in select patient populations, while still necessitating further robust comparative evidence across diverse cancer sites [89]. Despite these advantages, definitive evidence of broad clinical superiority over modern photon techniques remains indication-specific. While dosimetric benefits are consistently demonstrated, translation into improved survival or reduced toxicity outcomes depends strongly on tumor site, treatment volume, and patient selection. Randomized phase III evidence remains limited in several common disease settings, and uncertainties related to RBE variability, LET-based planning, and dose accumulation in adaptive proton therapy require further standardization before widespread biologically optimized implementation can be realized. Emerging developments—including intensity-modulated proton therapy, adaptive workflows, image guidance integration, compact accelerator systems, and ultra-high dose rate (FLASH) proton therapy—illustrate the rapid evolution of the field. A schematic representation of the proton therapy gantry head and its beam delivery components is shown in Figure 5. However, continued translational radiobiology research, rigorous clinical trials, and health economic evaluation will be essential to define which patient populations derive the greatest net benefit. Taken together, proton therapy should be viewed not as a universally superior alternative to photon radiotherapy, but as a precision modality with clear physical advantages and evolving bio-logical rationale whose optimal clinical role continues to be refined.

## Figures and Tables

**Table 1:** Characteristics of simple versus complex (clustered) DNA damage induced by low- vs. high-LET radiation based on the current state of knowledge.

Feature	Low-LET (Photons)	High-LET (Protons/Carbon Ions at Distal SOBP)
Lesions per track	Isolated	Multiple (≥2 DSBs + SSBs + base damage)
Spatial scale	>10–20 nm apart	Within 1–2 DNA helical turns (<10 nm)
Repair pathways affected	Base Excision Repair (BER), NHEJ, HR (efficient)	Overwhelmed NHEJ and HR → persistent damage
Biological consequence	Repairable, sublethal	Lethal, reduced shoulder on survival curve
RBE (typical clinical range)	1.0	1.8–5.1 (protons), 2–5 (carbon ions)

**Table 2:** Representative proton LET and range values derived from Monte Carlo DNA damage simulations reported by Nikitaki et al. [9].

Energy	100 keV	250 MeV
LET	76.77 keV μm^−1^	0.394 keV μm^−1^
Range in H_2_O	1.62 μm	37.98 cm

**Table 3:** Illustrative yields of double-strand breaks (DSBs) and total DNA damage clusters per Gy·Gbp based on Monte Carlo simulations reported by Nikitaki et al. [9].

	DSB/Gy·Gbp		Clusters/Gy·Gbp	
Energy	100 keV	250 MeV	100 keV	250 MeV
p O_2_ = 0.1%	25.40 ± 0.03	5.98 ± 0.02	208.00 ± 0.06	55.4 ± 0.1
p O_2_ = 20%	25.82 ± 0.03	8.12 ± 0.02	206.91 ± 0.06	61.9 ± 0.1

**Table 4:** Illustrative fraction of complex DNA damage and number of clusters per cell per track based on Monte Carlo simulations reported by Nikitaki et al. [9].

	Fraction of Complex Damage (DSBs and Base Damage Included)		Number of DNA Damage Clusters per Cell per Track	
Energy	100 keV	250 MeV	100 keV	250 MeV
p O_2_ = 0.1%	95.06%	45.57%	6.063 ± 0.008	(1.925 ± 0.007) × 10^−2^
p O_2_ = 19.4%	95.17%	51.76%	6.163 ± 0.008	(2.619 ± 0.007) × 10^−2^

**Table 5:** Comparison of proton therapy and photon IMRT across key dosimetric, biological, and clinical features.

Feature	Proton Therapy	Photon IMRT
Depth–Dose Distribution	Characterized by Bragg peak with minimal exit dose	Delivers dose along entire beam path, including exit dose
Dose Conformity	Highly conformal in depth; lateral conformity depends on technique (e.g., IMPT)	Highly conformal with advanced modulation techniques (IMRT/VMAT)
Normal Tissue Exposure	Often reduces integral dose and exposure to distal organs; magnitude is indication-dependent	Greater integral dose due to entrance and exit dose
Acute Toxicity	Frequently reduced in selected indications (e.g., pediatric, CNS, mediastinal tumors); not universal	Well-characterized toxicity profile; comparable in some disease sites
Late Toxicity	Potential reduction in long-term sequelae due to reduced integral dose; site-specific	Established long-term data; risk depends on treated volume and location
Pediatric Applications	Often preferred due to reduction in secondary malignancy risk and growth-related toxicity	Used when proton access is limited or benefit is uncertain
Relative Biological Effectiveness (RBE)	Typically assumed 1.1 clinically; may vary with LET and depth	Defined as 1.0
Infrastructure and Cost	Higher capital investment; facility-level economics vary	Lower capital investment; widely available
Clinical Evidence Base	Growing prospective data; limited randomized phase III evidence in common adult tumors	Extensive long-term randomized evidence base

## Data Availability

All necessary data and information exist in the manuscript. Further details may be requested from the authors.
